# Quality of life, fatigue and local response of patients with unstable spinal bone metastases under radiation therapy - a prospective trial

**DOI:** 10.1186/1748-717X-9-133

**Published:** 2014-06-11

**Authors:** Harald Rief, Maximiliane Heinhold, Thomas Bruckner, Ingmar Schlampp, Robert Förster, Thomas Welzel, Tilman Bostel, Jürgen Debus, Stefan Rieken

**Affiliations:** 1 Department of Radiation Oncology, University Hospital of Heidelberg, Im Neuenheimer Feld 400, 69120 Heidelberg, Germany; 2 Department of Medical Biometry, University Hospital of Heidelberg, Im Neuenheimer Feld 305, 69120 Heidelberg, Germany

**Keywords:** Bone metastases, Spine, Stability, Unstable metastases, Palliative radiotherapy

## Abstract

**Background:**

To evaluate the local response according to stability after radiotherapy (RT) with a special focus on quality-of-life (QoL), fatigue, pain and emotional distress in patients with unstable spinal bone metastases.

**Methods:**

In this prospective trial, 30 patients were treated from September 2011 until March 2013. The stability of osteolytic metastases in the thoracic and lumbar spine was evaluated on the basis of the Taneichi-score after three and six months. EORTC QLQ-BM22, EORTC QLQ-FA13, and QSC-R10 were assessed at baseline, and three months after RT.

**Results:**

After 3 months, 25% (n = 6) and after 6 months 33.3% (n = 8) were classified as stable. QoL, fatigue, and emotional distress showed no difference over the course. The pain response 3 months after RT showed a significant difference (p < 0.001). Pathological fractures occurred in 8.3% of the patients (n = 2) within six months following RT.

**Conclusions:**

Our trial demonstrated that RT can improve stability in one third of patients over a 6-months period with unstable spinal metastases. Importantly, for these patients pain relief was detected but RT had no impact on QoL, fatigue, and emotional distress.

**Trial registration:**

Clinical trial identifier NCT01409720.

## Introduction

Spinal bone metastases represent the most frequent site of skeletal metastasis [1]. The effects of bone metastases are a major clinical concern and result in pain at rest and during exercise, limitations in daily life, lower performance ability, risk of pathological fractures and neurologic deficits [2]. Radiotherapy (RT) is the most common treatment option of bone metastases in advanced tumor disease [3]. Standard clinical care of unstable metastases often includes patient immobilization either by means of an orthopedic thoracic corset or by confining the patient to bed in order to prevent pathological fractures, which further decreases patients’ quality-of-life (QoL). Fatigue is one of the most prevalent and distressing symptoms reported by cancer patients [4]. For these reasons, local control, and re-ossification of formerly unstable osteolytic lesions are an important clinical challenge and represent a goal of any therapy aiming to manage osteolytic bone metastases. An essential aspect is the stability of the vertebral bodies affected. A recent trial showed that the use of a systematic radiological scoring system to classify osteolytic metastases of the vertebral column was practicable in daily routine [5].

The effect of palliative RT in patients with unstable bone metastases is not well analyzed. The aim of this prospective trial was to evaluate the local response according to stability after RT with a special focus on QoL, fatigue, pain and emotional distress in patients with unstable spinal bone metastases.

## Methods

**Figure 1:**
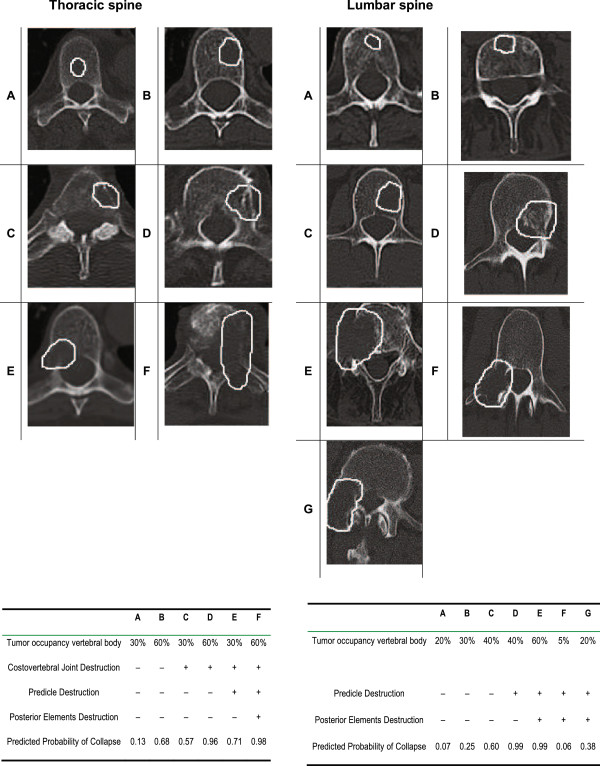
Taneichi score.

From September 2011 through March 2013, 30 consecutive patients with a histologically confirmed cancer of any primary and unstable spinal osteolytic metastases of the thoracic or lumbar segments were screened and included at the Radiooncology Department of the Heidelberg University Clinic. Inclusion criteria were an age of 18 to 80 years, written consent to participate, and already initiated bisphosphonate therapy. The patients were subjected to a staging of their vertebral column within the context of the computed tomography (CT) designed to plan the radiation schedule prior to enrolment into the trial. In this examination metastases were classified as “stable” or “unstable”. Patients with unstable vertebral-body lesions were included. The stability of each affected vertebral body was assessed according to the Taneichi score [6] on the basis of the CT image recorded before RT and also during follow-up restaging CT’s three and six months after RT (Figure 1).

The osteolytic metastases were rated on a scale from A to F. Subtypes A to C were defined as stable, subtypes D to F as unstable. In cases in which only one lesion was rated as unstable in a patient with multiple metastases, the score was “unstable”. The stability was measured after twelve weeks, and after six months.

The target parameters of QoL, fatigue, and emotional distress were assessed at baseline (t_0_) and twelve weeks after RT (t_2_), and comprise the documentation and completion of the questionnaires EORTC QLQ-BM22, EORTC QLQ-FA13, QSC-R10, and the recording of patient-specific data. For pain documentation, we used the visual analog scale. The “bone survival” was defined as time from initial diagnosis of spinal bone metastases to death. The start of irradiation of bone metastases was not equalized to the initial diagnosis of bone metastases. Bone metastases distant from the irradiated site were not included. Overall survival was defined as time from initial diagnosis of primary site to death. We estimated patient survival using the Kaplan-Meier survival method. Patients were censored on the basis of whether they were alive. The data of the patient records was collected by the authors. Patient characteristics are shown in Table 1. The study was approved by the Heidelberg Ethics Committee (Nr. S-316/2011).

**Table 1:** Patient characteristics at baseline

	**n**	**%**
Age (years)		
Mean (SD)	64.3 (9.6)	
Gender		
Male	20	66.7
Female	10	33.3
Karnofsky PS (median, range)	80 (60–90)	
Primary site		
Lung	14	46.7
Breast	7	23.3
Prostate	3	10.0
Kidney	1	3.3
Other	5	16.7
Site of spinal metastases		
Thoracic	18	60.0
Lumbar	12	40.0
Number metastases		
Mean (range)	2.8 (1–11)	
Solitary	13	43.3
Multiple	17	56.7
Concomitant metastases at baseline		
Visceral	12	40.0
Brain	3	10.0
Lung	7	23.3
Tissue	8	26.7
Surgical corset	16	53.3
Bisphosphonates before RT	28	93.3
Antihormonal therapy before RT	3	10.0
Chemotherapy before RT	20	66.7
Pathological fractures at baseline	4	13.3

SD Standard deviation, RT Radiotherapy.

### Radiotherapy

RT was performed at the Radiooncology Department of the Heidelberg University Clinic. After virtual simulation was performed to plan the radiation schedule, RT was carried out via one single dorsal photon field using 6 MV photons. Primary target volume (PTV) covered the specific vertebral body affected as well as the ones immediately above and below. Twenty-five patients (83.3%) were treated with 10 × 3 Gy, five patients (16.7%) with 20 × 2 Gy. The median individual dose in all patients was 3 Gy (range 2–3 Gy), the median total dose 30 Gy (range 30–40 Gy). The individual and total doses were decided separately for each individual patient, depending on the histology, the patient’s general state of health, and on the current staging and the corresponding prognosis.

### Sample calculation and statistical analysis

The total number of patients undergoing RT in the radiation oncology department of the Heidelberg University Clinic for metastatic processes in the vertebral column in the recruitment period is approx. 120, about 60 of whom shall fulfill the inclusion criteria. On account of the explorative character of this study it was not possible to estimate the total number of cases; with a scheduled number of 30 patients, it will, however, be possible to detect a standardized effect (Cohen’s d) of 0.8 with a power of 80% and an α significance level of 5%. All variables were analyzed descriptively by tabulation of the measures of the empirical distributions. According to the scale level of the variables, means, and standard deviations or absolute and relative frequencies, respectively, will be reported. Descriptive p-values of the corresponding statistical tests comparing the treatment times will be given. Additionally, for difference between t_0_ and t_2_ Wilcoxon signed rank test was used. The Cohen’s effect (ES) size was assessed for clinically relevant changes in questionnaires measures (<0.3 low, 0.3-0.7 moderate, >0.7 strong difference). All statistical analyses were done using SAS software Version 9.1 (SAS Institute, Cary, NC, USA).

### Measures of primary and secondary End points

The primary endpoint was local response and assessed the stability according to Taneichi-score [6] over the course. Secondary endpoints were QoL, fatigue, emotional distress, and pain. QoL, assessed using the EORTC QLQ BM22 questionnaire, which is specially designed for patients with bone metastases. The QLQ BM22 module (range 0–100) comprises 22 items and four scales for the measurement of pain in various parts of the body (painful sites), pain characteristics (persistent pain, recurrent pain), functional impairment (occurrence of pain when performing different activities, interference with everyday activities), and psychosocial aspects (family, worries, hope) [7]. Fatigue was assessed using the EORTC QLQ FA13 (range 0–100). This QLQ FA13 module includes 13 items and five scales for measuring cancer-related fatigue [8], with subscales covering physical fatigue, emotional fatigue, cognitive fatigue, interference with daily life, and social sequelae. Emotional distress was assessed using the QSC-R10 (range 0–50) questionnaire. The QSC-R10 [9] module is a valid and reliable questionnaire for determining emotional distress and anxiety in cancer patients [10]. The questionnaires were filled out by the patients at the study site. Pain was assessed according the visual analog scale (0–100).

## Results

The median follow-up was 13.2 months. The survival status at last follow-up showed that 7 patients (23.3%) were still alive and 23 patients (76.7%) had died from cancer. Six patients (20.0%) died within the first twelve weeks following RT, no additional patient died within 6 months due to tumor progression.

The evaluation of the distribution of subtypes A to F (Table 2) showed a minor change in the direction of improvement over the course of time. After 3 months, improvement occurred in 33.3% of the cases (n = 8), no change was seen in 66.7% (n = 16) of the cases. After 6 months the distribution showed the same results of subtypes. No deterioration was seen after 3 and 6 months. This Bowker test shows the distribution pattern of the subtypes according to Taneichi prior to, three and six months after RT. Asymmetry was apparent (p = 0.238 and p = 0.629) and the correlation (kappa = 0.55 and 0.57) was good (Table 2). According to Taneichi-score, after 3 months 25% (n = 6) and after 6 months, 33.3% (n = 8) were classified as stable (Table 3).

**Table 2:** The evaluation of the distribution of subtypes stable and unstable metastases over the course of time (0–3 and 0–6 month)

**A. Subtypes after 3 months**								
Subtypes before radiotherapy		**A**	**B**	**C**	**D**	**E**	**F**	**Total**
	**A**	**0**	0	0	0	0	0	0
	**B**	0	**0**	0	0	0	0	0
	**C**	0	0	**0**	0	0	0	0
	**D**	0	0	2	**5**	0	0	7
	**E**	0	0	3	2	**6**	0	11
	**F**	0	0	1	0	0	**5**	6
	**Total**	0	0	6	7	6	5	**24**
**B. Subtypes after 6 months**								
Subtypes before radiotherapy		**A**	**B**	**C**	**D**	**E**	**F**	**Total**
	**A**	**0**	0	0	0	0	0	0
	**B**	0	**0**	0	0	0	0	0
	**C**	0	0	**0**	0	0	0	0
	**D**	0	0	2	**5**	0	0	7
	**E**	0	1	4	0	**6**	0	11
	**F**	0	0	1	0	0	**5**	6
	**Total**	0	1	7	5	6	5	**24**

This Bowker Test showed the distribution of subtypes of Taneichi-Score before and 3 months after radiation therapy (A). Asymmetry was apparent (p = 0.238) and the correlation (kappa = 0.55) was good. The evaluation of the distribution of subtypes A to F showed a minor change in the direction of improvement over the course of time. Deterioration occurred in 0% of the cases, improvement in 33.3% (n = 8). No change was seen in 66.7% (n = 16) of the cases. After 6 months (p = 0.629, kappa = 0.57) the distribution showed the same results of subtypes.

**Table 3:** The results of Taneichi Score evaluation

	**n**	**%**
Stability after 3 months		
Unstable	18	75.0
Stable	6	25.0
Stability after 6 months		
Unstable	16	66.7
Stable	8	33.3
Bone fracture before RT		
Yes	4	13.3
No	26	86.7
Bone fracture 6 months after RT		
Yes	6	25.0
No	18	75.0

Stability after 3 and 6 months, pathological fracture at baseline and 6 months after RT.

In QoL according to the BM22, fatigue according to the FA13, and emotional distress according to the QSC-R10, no difference was seen after 3 months (Tables 4, 5, 6). The pain response 3 months after RT showed a significant difference (P < 0.001, ES 0.83) (Table 6).

**Table 4:** Effects of RT on quality of life (EORTC QLQ-BM 22)

**Symptom scales**	**n**	**mean**	**SD**
**Painful sites**			
baseline (t0)	30	42.00	26.27
3 months (t2)	24	36.67	24.48
Treatment effect (t0-t2) after 3 months p = 0.346			
Effect size (t0-t2) after 3 months = 0.21			
**Pain characteristics**			
baseline (t0)	30	47.04	33.10
3 months (t2)	24	39.35	34.67
Treatment effect (t0-t2) after 3 months p = 0.172			
Effect size (t0-t2) after 3 months = 0.33			
**Functional interference**			
baseline (t0)	30	53.06	28.17
3 months (t2)	24	46.18	28.55
Treatment effect (t0-t2) after 3 months p = 0.186			
Effect size (t0-t2) after 3 months = 0.26			
**Psychosocial aspects**			
baseline (t0)	30	60.19	21.50
3 months (t2)	24	56.48	21.2
Treatment effect (t0-t2) after 3 months p = 0.082			
Effect size (t0-t2) after 3 months = 0.34			

**Table 5:** Effects of RT on fatigue (EORTC QLQ-FA 13)

**Symptom scales**	**n**	**mean**	**SD**
**Physical fatigue**			
baseline (t0)	30	55.00	32.06
3 months (t2)	24	51.39	31.63
Treatment effect (t0-t2) after 3 months p = 0.534			
Effect size (t0-t2) after 3 months = 0.04			
**Emotional fatigue**			
baseline (t0)	30	36.94	30.14
3 months (t2)	24	29.17	30.00
Treatment effect (t0-t2) after 3 months p = 0.616			
Effect size (t0-t2) after 3 months = 0.12			
**Cognitive fatigue**			
baseline (t0)	30	21.48	25.59
3 months (t2)	24	20.83	24.37
Treatment effect (t0-t2) after 3 months p = 0.304			
Effect size (t0-t2) after 3 months = 0.23			
**Interference with daily life**			
baseline (t0)	30	50.19	31.70
3 months (t2)	24	48.47	30.21
Treatment effect (t0-t2) after 3 months p = 0.563			
Effect size (t0-t2) after 3 months = 0.10			
**Social sequelae**			
baseline (t0)	30	54.19	51.50
3 months (t2)	24	52.48	50.21
Treatment effect (t0-t2) after 3 months p = 0.583			
Effect size (t0-t2) after 3 months = 0.11			

**Table 6:** Pain over the course and emotional distress according to QSC-R10 questionnaire

	**n**	**mean**	**SD**
**QSC-R10**			
baseline (t0)	30	21.90	10,81
3 months (t2)	24	18.88	9,25
Treatment effect (t0-t2) after 3 months p = 0.108			
Effect size (t0-t2) after 3 months = 0.29			
**Visual analog scale**			
baseline (t0)	30	51.7	20.0
3 months (t2)	24	40.7	22.0
Treatment effect (t0-t2) after 3 months p < 0.001			
Effect size (t0-t2) after 3 months = 0.83			

**Figure 2:**
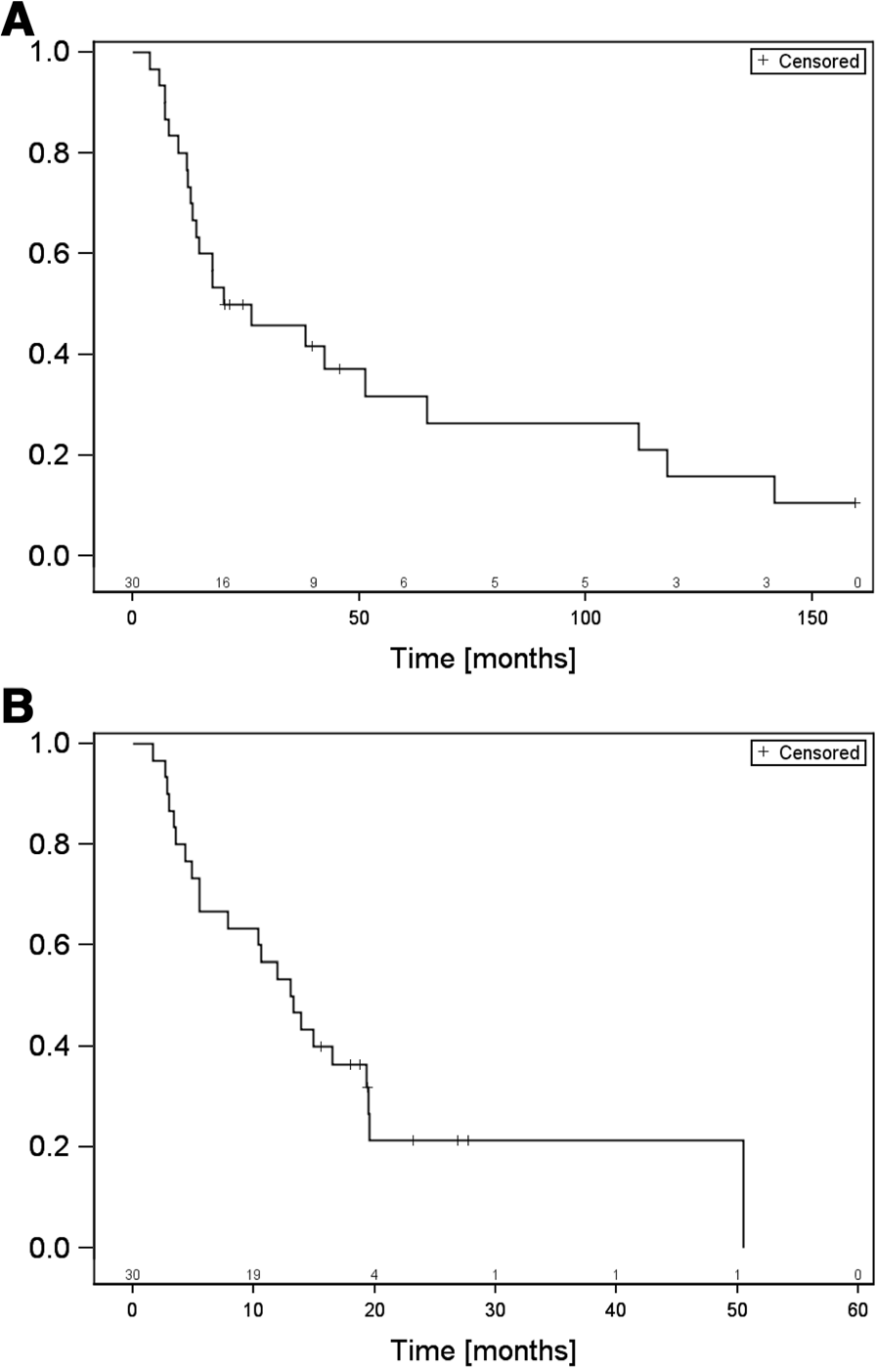
**A. Overall survival, B. Bone survival.** The numbers of patients at risk are mentioned above the time in months.

A pathological fracture was diagnosed in 4 patients (13.3%) prior to RT. Fractures occurred in 8.3% of the patients (n = 2) within six months following RT (Table 3).These data correspond to a six-months overall survival of 93.3%, a one-year survival of 76.7%, and a two-year survival of 45.8% (Figure 2A). The “bone survival” was 66.7% at six months, 53.3% at one year, and 26.6% at two years (Figure 2B).

## Discussion

Bone metastases frequently occur in advanced cancer diseases, and the majority of cases are localized in the spinal column. Re-calcification of unstable lesions is one of the main goals of palliative RT. Patients affected are in most cases immobilized, primarily due to the risk of pathological fracture and the associated risk of spinal cord compression. An early identification of osteolytic metastases and their classification in terms of stability is a major factor in the decision for the therapeutical measures to be taken. The scoring system according to Taneichi [6] constitutes a simple method for classifying the vertebral bodies as “stable” or “unstable”, which is why this score is employed in this evaluation. Here, we show regained stabilization due to RT-induced re-calcification in 25% and 33% of patients after 3 and 6 months, respectively. Rief et al. [5] described the therapeutic response of osteolytic spinal lesions in lung cancer following RT. Of the 123 patients in whom the metastases were classified unstable prior to radiotherapy, 21 patients (17%) were classified stable after three months, and 30 patients (24%) stable after six months. Our calculation was higher due to different histologies and smaller sample size. However, RT and consecutive re-calcification of the lesions made it possible to classify 33.3% of the originally unstable osteolytic processes as stable after six months. Our results showed a pathological fracture in 13.3% of the vertebral bodies prior to RT. Other pathological fractures up to six months in the further course were seen only in 2 cases (8.3%). In previous retrospective studies among American and Japanese populations, the incidence of pathologic fractures in the vertebral column is given at 10% [11,12] and was comparable with our data.

No substantial improvement with small to moderate effect sizes was observed in all dimensions of the EORTC QLQ-BM 22 (painful sites, pain characteristics, functional interference, and psychosocial aspects) after three and six months. With a high rate of local response and stability due to RT, patients with unstable metastases would benefit from RT. Through the low local response and small sample size, we were not able to show any difference in QoL. Lam et al. showed that baseline KPS, age, and employment status had significant impacts in self-reported HRQOL in patients with bone metastases receiving palliative RT [13]. However, we could not show predictive factors due to our small sample size. Fatigue (EORTC QLQ-FA13), and emotional distress (QSC-R10) showed no statistical significance between baseline and after 3 months as well. Cancer-related fatigue is defined as a persistent subjective sensation of tiredness relating to cancer treatment that impairs the patients’ physical and mental performance [14]. It is of great clinical relevance for patients in an advanced stage of cancer disease [15]. RT determined a therapy effect in pain: the pain score after three months was smaller (p < 0.001, ES 0.83) than at baseline. Palliative RT is an established effective modality for the treatment of pain in patients with bone metastases of the spinal column [16,17] and will continue to remain the principal option for the treatment of painful bone metastases [18].

The survival of patients with bone metastases is still poor. In a recent study, Rief et al. [19] showed that the stability of the vertebral bodies played no role with regard to survival. Our results underlined the very low survival times in patients with unstable metastases.

Limitations of the study are the relatively small sample size, the variety of primary tumors and patient conditions, and the exclusion of patients presenting with cervical spine metastases. Questionnaires were only provided at baseline and 3 months after RT - as this time period was believed to be sufficient to demonstrate clinical changes. We lack systematic knowledge of possible further improvement after 6 and 12 months. Among the strengths of the study are the prospective setting, the use of a scoring system for stability of bone metastases and standardized and specific measures to assess multiple domains of QoL among patients with unstable bone metastases.

## Conclusion

In this group of patients with unstable bone metastases we were able to show that RT can improve stability in one-third of patients over a 6-months period. Importantly, these patients showed a pain response after 3 months, but RT alone is not sufficient enough to improve QoL and fatigue, and reduce emotional distress and anxiety specific to patients suffering from spinal metastases. Large controlled trials are necessary to confirm these findings.

## Competing interests

The authors declare that they have no competing interests.

## Authors’ contributions

HR, SR and JD developed and planned this trial. TB was responsible for statistical considerations/basis of the analysis. TW estimated the stability of bone metastases. HR, MH, RF, IS, and SR performed the examinations and RT supervisions. HR and MH made the data collection. All authors read and approved the final manuscript.

## References

[B1] Coleman RE Metastatic bone disease: Clinical features, pathophysiology and treatment strategies Cancer Treat Rev 2001 27 165 176 10.1053/ctrv.2000.0210 11417967

[B2] Wyne CM Hu SS Lotz JC Biomechanically derived guidline equations for burst fracture risk prediction in the metastatically involved spine J Spin Disord Tech 2003 16 2 180 185 10.1097/00024720-200304000-00010 12679673

[B3] Gerszten PC Welch WC Current surgical management of metastatic spinal disease Oncology 2000 14 1013 1036 10929589

[B4] Ahlberg K Ekman T Gaston-Johansson F Mock V Assessment and management of cancer-related fatigue in adults Lancet 2003 362 640 650 10.1016/S0140-6736(03)14186-4 12944066

[B5] Rief H Bischof M Bruckner T Welzel T Askoxylakis V Rieken S Lindel K Combs S Debus J The stability of osseous metastases of the spine in lung cancer–a retrospective analysis of 338 cases Radiat Oncol 2013 8 1 200 10.1186/1748-717X-8-200 23937907 PMC3751223

[B6] Taneichi H Kaneda K Takeda N Abumi K Satoh S Risk factors and probability of vertebral body collapse in metastases of the thoracic and lumbar spine Spine 1997 22 239 245 10.1097/00007632-199702010-00002 9051884

[B7] Chow E Nguyen J Zhang L Tseng LM Hou MF Fairchild A Vassiliou V Jesus-Garcia R Alm El-Din MA Kumar A Forges F Chie WC Bottomley A International field testing of the reliability and validity of the EORTC QLQ-BM22 module to assess health-related quality of life in patients with bone metastases Cancer 2012 3 1457 1465 21837676 10.1002/cncr.26410

[B8] Weis J Arraras JI Conroy T Efficace F Fleissner C Görög A Hammerlid E Holzner B Jones L Lanceley A Singer S Wirtz M Flechtner H Bottomley A Development of an EORTC quality of life phase III module measuring cancer-related fatigue (EORTC QLQ-FA13) Psychooncology 2013 22 5 1002 1007 10.1002/pon.3092 22565359

[B9] Book K Marten-Mittag B Henrich G Dinkel A Scheddel P Sehlen S Haimerl W Schulte T Britzelmeir I Herschbach P Distress screening in oncology-evaluation fo the questionaire on distress in cancer patients-short form (QSC-R10) in a German sample Psycho-Oncology 2011 20 287 293 10.1002/pon.1821 20669340

[B10] Escalante CP Treatment of cancer-related fatigue: an update Support Care Cancer 2003 11 79 83 12560935 10.1007/s00520-002-0406-8

[B11] Kosteva J Langer CJ Incidence and distribution of skeletal metastases in NSCLC in the era of PET Lung Cancer 2004 46 45

[B12] Tsuya A Kurata T Tamura K Fukuoka M Skeletal metastases in non-small cell lung cancer: A retrospective study Lung Cancer 2007 57 229 232 10.1016/j.lungcan.2007.03.013 17451841

[B13] Lam K Chow E Zhang L Wong E Bedard G Fairchild A Vassiliou V El-Din MA Jesus-Garcia R Kumar A Forges F Tseng LM Hou MF Chie WC Bottomley A Determinants of quality of life in advanced cancer patients with bone metastases undergoing palliative radiation treatment Support Care Cancer 2013 21 11 3021 3030 10.1007/s00520-013-1876-6 23775156

[B14] Mock V Atkinson A Barsevick A Cella D Cimprich B Cleeland C Donnelly J Eisenberger MA Escalante C Hinds P Jacobsen PB Kaldor P Knight SJ Peterman A Piper BF Rugo H Sabbatini P Stahl C NCCN Practice Guidelines for cancer-related fatigue Oncology 2000 14 151 161 11195408

[B15] Mock V Dow KH Meares C Grimm PM Dienemann JA Haisfield-Wolfe ME Quitasol W Mitchell S Chakravarthy A Gage I Effects of exercise on fatigue, physical functioning and emotional distress during radiation therapy for breast cancer Oncol Nurs Forum 1997 24 991 1000 9243585

[B16] Chow E Harris K Fan G Tsao M Sze WM Palliative radiotherapy trials for bone metastases: A systemic review J Clin Oncol 2007 25 1423 1436 10.1200/JCO.2006.09.5281 17416863

[B17] Klimo P Jr Kestle JR Schmidt MH Clinical trials and evidence-based medicine for metastatic spine disease Neurosurg Clin N Am 2004 15 549 564 10.1016/j.nec.2004.04.016 15450889

[B18] Lutz S Berk L Chang E Chow E Hahn C Hoskin P Howell D Konski A Kachnic L Lo S Sahgal A Silverman L von Gunten C Mendel E Vassil A Bruner DW Hartsell W Palliative radiotherapy for bone metastases: An ASTRO evidence-based guideline Int J Rad Oncol Biol Phys 2011 79 4 965 976 10.1016/j.ijrobp.2010.11.026 21277118

[B19] Rief H Muley T Bruckner T Welzel T Rieken S Bischof M Lindel K Combs SE Debus J Survival and prognostic factors in non-small cell lung cancer patients with spinal bone metastases: a retrospective analysis of 303 patients Strahlenther Onkol 2014 190 1 59 63 10.1007/s00066-013-0431-1 24052009

